# The art of sleep: examining sleep strategies in the general population with a focus on the use of music for sleep

**DOI:** 10.1111/jsr.70006

**Published:** 2025-02-19

**Authors:** Rasmus Møller Buus, Silvia Genovese, Kira Vibe Jespersen

**Affiliations:** ^1^ Faculty of Health Aarhus University Aarhus Denmark; ^2^ Center for Music in the Brain, Department of Clinical Medicine Aarhus University & The Royal Academy of Music Aarhus/Aalborg Aarhus Denmark

**Keywords:** health behaviour, insomnia, music, sleep, sleep strategies, survey

## Abstract

Insomnia is highly prevalent and associated with serious health issues. Despite the negative impact of poor sleep on mind and body, most people do not seek treatment. This study aimed to examine the strategies used to improve sleep in the general population with a particular focus on music. A survey was distributed via Facebook advertisements to Danish adults aged ≥18 years. Among 3667 responders, a representative subsample of 1195 participants was randomly selected based on age and sex. Data analysis involved chi‐square tests and logistic regression with adjustments for age, sex, education, and insomnia status. The results showed that nearly all participants utilised strategies to aid their sleep, with the most common strategies being ‘following a routine’ (73%), ‘reducing caffeine in the afternoon/evening’ (65%) and ‘lowering the temperature in the bedroom’ (62%). Individuals with insomnia utilised significantly more strategies (average 8.4 strategies) compared to those without insomnia (average 6.6 strategies). Among all participants, 20% reported using music for sleep at least 1–2 times/month, whereas 11% used music for sleep weekly, and 4.5% used music almost daily. Music users tended to be younger, and they were also more likely to have insomnia. By evaluating 24 different sleep strategies, this study shows that people do several things to promote sleep in their daily lives. These findings enhance our understanding of sleep behaviour, providing an important fundament for targeting future public health interventions to reduce the use of sleep disruptive strategies and promote strategies known to improve sleep health.

## 1 INTRODUCTION

Sleep is very important for our health and well‐being. Poor sleep and insomnia are related to multiple health problems including cardiovascular disease, mental health problems, and a reduced quality of life (Riemann et al., 2017; Wu et al., 2023). Nevertheless, sleep problems are highly prevalent, with around one third of the general population experiencing insomnia symptoms (Morin & Jarrin, 2022). In Denmark, recent studies show that >40% of adults report that they rarely or never/almost never get enough sleep to feel rested (Jensen, 2021; Møller et al., 2023).

Two of the most effective and utilised treatments for insomnia are cognitive behavioural therapy for insomnia (CBT‐I) and pharmacotherapy with benzodiazepines (Riemann et al., 2017). CBT‐I consists of psychoeducation/sleep hygiene, relaxation training, stimulus control therapy, sleep restriction therapy, and cognitive therapy (Riemann et al., 2023), and has been shown to have a comparable effect to pharmacotherapy in the short term, but to be superior in the long term (Mitchell et al., 2012). However, CBT‐I can be costly and time‐consuming as it often involves face‐to‐face treatment (Riemann et al., 2017).

Sleep medications, such as benzodiazepines, although being effective treatments in the management of insomnia, pose issues in the form of side effects, tolerance development, and dependence (Buscemi et al., 2007). Consequently, they are only recommended for shorter periods of treatment. Given these limitations in the available treatments, many people with sleep problems and insomnia often go undiagnosed and untreated (Brown et al., 2017; Morin et al., 2006), and when professional treatment is initiated, the choice is often medicinal (Jennum et al., 2015).

Instead of seeking professional help, many people use various self‐help strategies and rituals to improve their sleep. Previous studies have shown different results in how common self‐help strategies for sleep are, as well as differences in what sleep strategies are being used (Aritake‐Okada et al., 2009; Bjorvatn, Waage, & Saxvig, 2023; Morin et al., 2006; Urponen et al., 1988). For example, a population study from Japan evaluated non‐pharmacological self‐management practices for good sleep and found that the most common strategy was having a bath, reported by ~60% of the participants (Aritake‐Okada et al., 2009). Similarly, an epidemiological study from Canada focused on self‐help strategies for insomnia and found that reading was the most common strategy (Morin et al., 2006). In a recent Norwegian study, researchers investigated if people used tricks or methods to fall sleep and found that 34% of the representative sample reported using a strategy, with relaxation exercises being the most common one (Bjorvatn, Waage, & Saxvig, 2023). Furthermore, this study found that people with insomnia tended to use more strategies than people without. This variation in the results may be due to different methodological approaches as well as cultural differences. Knowledge on sleep strategies is important to understand sleep behaviour in the general population and can be used to inform public health interventions. Here, we took a comprehensive approach to evaluate a broad selection of strategies to improve sleep, including the frequency of use.

One common sleep strategy found across the globe is listening to music (Brown et al., 2017; Morin et al., 2006; Scarratt et al., 2023; Trahan et al., 2018). Research suggests that music can exert a positive effect on human health, lessening stress and anxiety and stimulating the perception of pleasure, reward and motivation (Jespersen, Gebauer, & Vuust, 2022; Viola et al., 2023). Building on this, we know that listening to music may have a beneficial effect on subjective measures of sleep quality in people with insomnia (Jespersen, Pando‐Naude, et al., 2022; Wang et al., 2021). A study investigating the sleep strategies of the Canadian population found that 25.2% had used music for sleep within the last year (Morin et al., 2006). However, the study did not investigate this prevalence further, nor the characteristics of people using music for sleep. Another study suggests that people using music are characterised by being younger (Aritake‐Okada et al., 2009; Trahan et al., 2018) and more likely to have insomnia (Trahan et al., 2018). Still, the knowledge on music use for sleep is limited as no studies have explored how regularly people use music for sleep, nor the characteristics of music users in a population sample. The development of streaming services and smartphones has significantly expanded access to music, creating new opportunities for its use as a sleep‐promoting strategy. Given that music is a non‐pharmacological, accessible, and safe intervention, further research on its use for sleep is highly relevant. Clinical studies have already demonstrated its beneficial effects on sleep quality. Understanding how frequently people use music for sleep, as well as identifying the characteristics of these users, could inform the development of future targeted public health initiatives.

The aim of this study was to map the prevalence of a broad range of strategies used to aid sleep in the general population as well as their frequency of use. In addition, we aimed to characterise people who use music as a strategy. Based on previous studies, we hypothesized that people with insomnia would use more strategies than people without insomnia (Bjorvatn, Waage, & Saxvig, 2023). Furthermore, we expected that people who use music for sleep are younger and more likely to have insomnia (Aritake‐Okada et al., 2009; Trahan et al., 2018).

## 2 METHODS

### 2.1 The survey

The study was approved by the Institutional Review Board at Aarhus University (2023‐015). To investigate the sleep strategies in the general population, we developed an online survey using SurveyXact (Rambøll, 2024) provided by Aarhus University. The survey was distributed through Facebook advertisements to the Danish population aged ≥18 years, with the goal of reaching a sample of 1000 participants, representative of age and sex. Facebook is the most common social media in Denmark, used by 84% of the population (Statistikbanken, 2024).

Demographic and socioeconomic data were collected including age, sex, income range, educational level, nationality, and municipality of residence. To assess insomnia symptoms, we used the Bergen Insomnia Scale (BIS) (Pallesen et al., 2008), while sleep quality was evaluated using the Pittsburgh Sleep Quality Index (PSQI) (Buysse et al., 1989). The full content of the survey is presented in Data S1, Table S1.

The survey covered the 24 most common different sleep strategies, selected from previous studies (Aritake‐Okada et al., 2009; Brown et al., 2017; Morin et al., 2006; Trahan et al., 2018; Urponen et al., 1988). The amount of included sleep strategies was selected balancing between including the most relevant strategies while keeping the list manageable for respondents. In the final version, the option ‘other strategies’ was included to make room for respondents to specify strategies not included in the survey. The main question ‘What do you do to improve your sleep?’ was formulated based on previous studies addressing the same question (Aritake‐Okada et al., 2009; Bjorvatn, Waage, & Saxvig, 2023; Brown et al., 2017; Morin et al., 2006; Trahan et al., 2018; Urponen et al., 1988). Additionally, the survey was tested in a pilot sample of 20 participants, who provided feedback on their comprehension of the questions and suggested additional sleep strategies to be included into the final version.

Respondents who reported using music as a sleep aid were presented with additional questions, including the Goldsmith Musical Sophistication Index (Gold‐MSI) – Active Engagement subscale (Møller et al., 2024; Müllensiefen et al., 2014) and part of the Ollen Musical Sophistication Index (Ollen, 2006) to assess their active engagement and integration of music into their lives.

### 2.2 Data processing and analysis

#### 2.2.1 Data preparation

The initial dataset contained 3664 respondents. Our reference for a representative sample was the most recent census (2023) of the Danish population obtained from ‘Danmarks Statistik’ (Danmarks Statestik, 2023). The representative dataset was prepared by removing participants who did not answer ‘Male’ or ‘Female’ in the question about sex, to match the categories from ‘Danmarks Statistik’ (Danmarks Statestik, 2023). Furthermore, participants answering ‘No’ to the question ‘Is your nationality Danish?’ were excluded, as the survey addressed the Danish population. Finally, participants aged <18 and >120 years were removed to exclude non‐adults and errors.

Using Rstudio (Team, R, 2024), the dataset was subsampled using the R packages ‘sampling’ and ‘samplingbook’ to achieve the largest possible random subsample (Manitz et al., 2021; Yves Tillé, 2021). This resulted in 1195 participants representative of the Danish population regarding age and sex (Data S1, Figure S1).

#### 2.2.2 Assessment of insomnia and sleep quality

Data on insomnia and sleep quality were collected utilising the BIS and PSQI, respectively. Similar to other studies (Bjorvatn, Waage, Buysse, & Saxvig, 2023; Bjorvatn, Waage, & Saxvig, 2023; Torsvik et al., 2023), we used an adapted version of the BIS to assess insomnia disorder according to the fifth edition of the *Diagnostic and Statistical Manual of Mental Disorders* (DSM‐5) criteria (American Psychiatric Association, 2013). This means that the rating was based on the last 3 months instead of the past month used in the original version of the BIS based on the DSM‐Fourth Edition, Text Revision criteria. The BIS consists of six items rated on an 8‐point Likert scale indicating the number of days/week the participant experiences a specific insomnia symptom, resulting in a total BIS score ranging from 0 to 42 points. Based on the DSM‐5 criteria, the scoring defined insomnia disorder as experiencing symptoms at least 3 days/week on at least one of the first three items, as well as on at least one of the last two items including impact on daily functioning. The PSQI was used to assess sleep quality. The scoring system provided by Buysse et al. (1989) was used, resulting in a total score ranging between 0 and 21. An inhouse R‐script at the Center for Music in the Brain (Aarhus University) was utilised for the scoring. The suggested cut‐off score of >5 to be considered as poor sleep was applied (Buysse et al., 1989).

#### 2.2.3 Data analysis

RStudio software was used to perform the analyses (Team, R, 2024). The association between insomnia and the number of sleep strategies used was analysed using linear regression. Associations between the use of music for sleep and different characteristics were initially explored using Pearson's chi‐square statistics. To further investigate the relationships identified by the chi‐square test, we performed crude and adjusted logistic regression analyses using responses to the question: ‘What do you do to improve sleep’, with the option ‘Listening to music’ as the dependent variable. The approach for the statistical analysis was partly inspired by a previous study conducted on the Norwegian population (Bjorvatn, Waage, & Saxvig, 2023). The significance level was set to 0.05.

## 3 RESULTS

### 3.1 Participant characteristics

Table 1 contains information about the characteristics of participants in both the initial and subsampled datasets. All further mentions of results will be addressing the subsampled dataset.

**TABLE 1:** Demographics and characteristics of the participants in the initial dataset and subsampled dataset.

Variable	Initial dataset (*N* = 3667)	Subsampled dataset (*n* = 1195)
Sex, *n* (%)		
Male	927 (25)	589 (49)
Female	2685 (73)	606 (51)
Other	52 (2)	0 (0)
Age (years), *n* (%)		
18–29	565 (15)	232 (19)
30–39	574 (16)	185 (16)
40–49	702 (19)	182 (15)
50–59	741 (20)	203 (17)
60–69	669 (18)	171 (14)
≥70	410 (11)	222 (19)
Education (Danish levels of education), *n* (%)		
Primary school	96 (3)	33 (3)
Upper secondary education	338 (9)	127 (11)
Vocational education	539 (15)	189 (16)
Bachelor	1435 (39)	428 (36)
Master	1110 (30)	359 (30)
PhD	146 (4)	62 (5)
Income before taxes (DKK), *n* (%)		
<100,000	305 (8)	108 (9)
100,001–250,000	725 (20)	253 (21)
250,001–400,000	922 (25)	278 (23)
400,001–600,000	906 (25)	265 (22)
600,001–800,000	280 (8)	100 (8)
800,001–1,000,000	89 (2)	43 (4)
>1,000,000	51 (1)	21 (2)
Do not know	91 (3)	26 (2)
Do not wish to answer	295 (8)	101 (8)
PSQI		
Score ≤5, *n* (%)	1534 (42)	541 (45)
Score >5, *n* (%)	2129 (58)	654 (55)
PSQI mean score	6.85	6.56
BIS		
No insomnia, *n* (%)	2258 (62)	773 (65)
Insomnia, *n* (%)	1406 (38)	422 (35)
BIS mean score	13.59	13.01

Abbreviations: BIS, Bergen Insomnia Scale; DKK, Danish Kroner; PSQI, Pittsburgh Sleep Quality Index.

The prevalence of insomnia in the subsample was found to be 35% using the BIS, while 55% of participants were found to have sleep problems according to the PSQI scores. In all, 35% of respondents had completed Master level education or higher, while 3% of respondents had no further education than primary school. We had participants from all 98 municipalities of Denmark. Most respondents were from Copenhagen, Aarhus, Aalborg, and Odense, corresponding to the four largest municipalities in Denmark. For a comprehensive overview see Figure S2 in Data S1.

### 3.2 Sleep strategies

This study found that 99.0% of respondents used at least one sleep strategy at least 1–2 times/month. The average number of sleep strategies utilised at least 1–2 times/month was 7.2 per respondent. Table 2 shows an overview of how commonly the sleep strategies were used (see also Data S1, Table S2). The most common strategy was found to be ‘Following a routine’ (73%) followed by ‘Reducing caffeine in the afternoon/evening’ (65%) and ‘Lowering the temperature in the bedroom’ (62%). The least used strategies were ‘Writing in a diary’ (6%), ‘Listening to white noise’ (6%) and ‘Smoking cigarettes’ (7%). In all, 19% of respondents stated using other sleep strategies, not included in the options. Reviewing the free‐text responses, the most popular additional strategies were listening to audiobooks, doing sudoku, using earplugs, utilising positive thinking, knitting, and drinking tea.

**TABLE 2:** Sleep strategies.

Sleep strategy, *n* (%)	Used at least 1–2 times/month^a^	Used at least 1–2 times/week	Used almost every day or every day
Following a routine	868 (73)	833 (70)	644 (54)
Reducing caffeine in the afternoon/evening	780 (65)	757 (63)	676 (57)
Lowering temperature in the bedroom	739 (62)	665 (56)	560 (47)
Using a phone (scrolling, reading, gaming)	636 (53)	544 (46)	302 (25)
Reading a book	627 (52)	445 (37)	257 (22)
Reducing blue light exposure	521 (44)	481 (40)	384 (32)
Having sex alone/masturbation	514 (43)	242 (20)	49 (4)
Doing exercise	484 (41)	395 (33)	77 (6)
Watching television or streaming	409 (34)	329 (28)	176 (15)
Doing breathing exercises	377 (32)	206 (17)	71 (6)
Having sex with a partner	367 (31)	152 (13)	6 (1)
Doing meditation or mindfulness	260 (22)	134 (11)	53 (4)
Listening to podcast	247 (21)	176 (15)	110 (9)
Listening to music	243 (20)	130 (11)	54 (5)
Drinking alcohol	235 (20)	133 (11)	31 (3)
Other, please specify	231 (19)	210 (18)	163 (14)
Taking a hot shower	224 (19)	115 (10)	32 (3)
Praying	170 (14)	119 (10)	86 (7)
Doing body scan	162 (14)	74 (6)	21 (2)
Using non‐prescription sleep medication	112 (9)	73 (6)	33 (3)
Using prescription sleep medication	106 (9)	79 (7)	49 (4)
Using a sleep mask	100 (8)	70 (6)	41 (3)
Smoking cigarettes	83 (7)	73 (6)	55 (5)
Listening to white noise	77 (6)	39 (3)	20 (2)
Writing in a diary	72 (6)	40 (3)	23 (2)

*Note*: participants were allowed to select more than one option. Respondents were presented with the response options ‘Never’, ‘1–2 times/year’, ‘1–2 times/month’, ‘1–2 times/week’, ‘≥3 times/week’, ‘Almost every day’ and ‘Every day’.

^a^ Was used to sort responses from most to least popular.

### 3.3 Sleep strategies and insomnia

**FIGURE 1:**
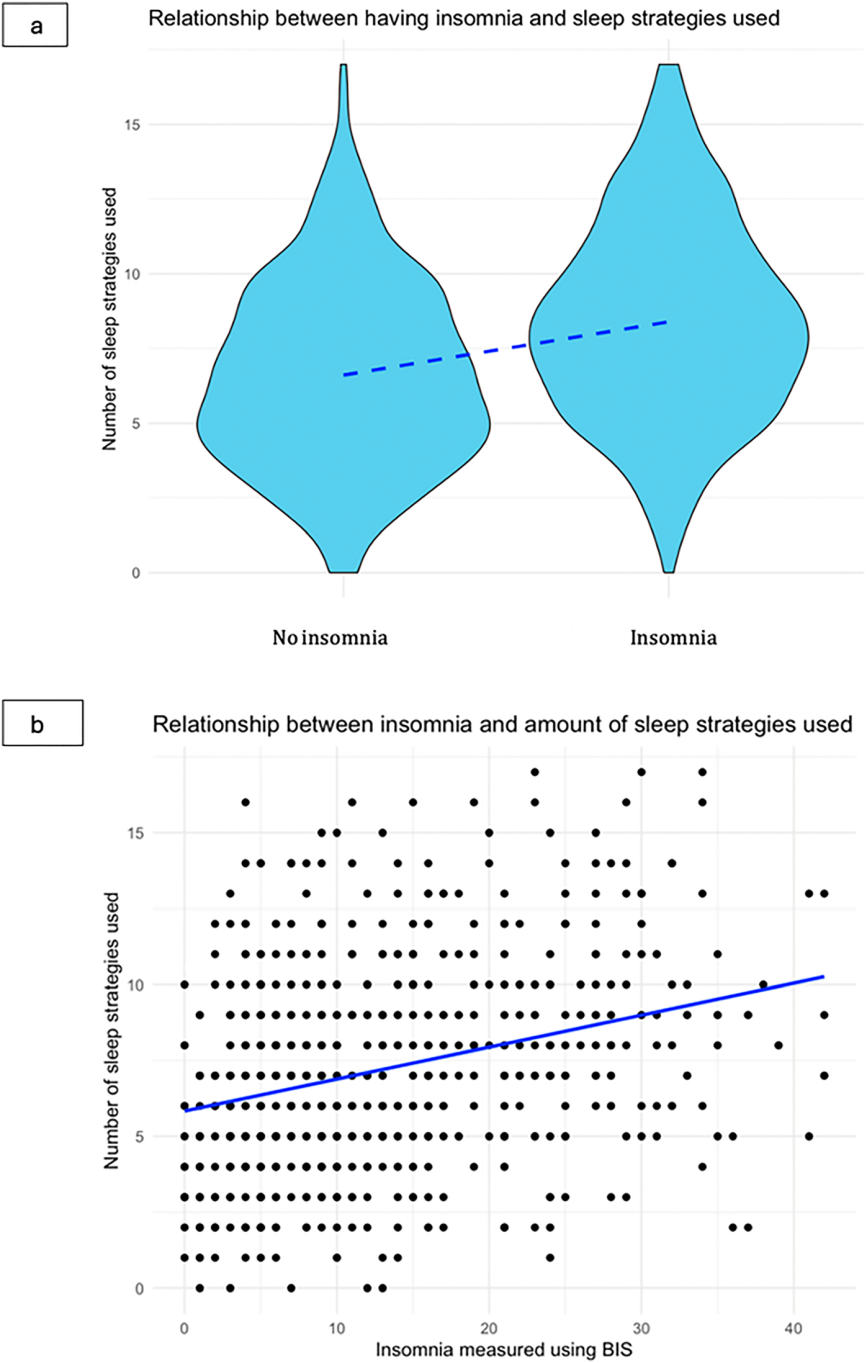
Insomnia and sleep strategies. Linear regression between the number of sleep strategies used at least 1–2 times/month and insomnia according to the fifth edition of the *Diagnostic and Statistical Manual of Mental Disorders* criteria measured with the Bergen Insomnia Scale (BIS). 1a is a violin plot of insomnia/no insomnia and number of sleep strategies used. 1b is a plot showing the severity of insomnia through participants’ BIS scores (ranging from 0 to 42) and the number of sleep strategies used.

People with insomnia used significantly more sleep strategies than people without insomnia. We found that people with insomnia used on average 8.4 different sleep strategies at least 1–2 times/month. People who did not have insomnia used 6.6 different sleep strategies at least 1–2 times/month. Calculating this difference using linear regression, we found the relationship to be highly significant with a *p* < 0.0001 and a coefficient of 1.79 (corresponding to the difference between 8.4 and 6.6 sleep strategies). However, the linear regression model had a *R* ^2^ value of 6.50%, meaning that only 6.5% of the variance in the number of sleep strategies is explained by insomnia. Performing linear regression using the numerical BIS scores, we found a coefficient of 0.10, indicating that with every 1‐point increase in the BIS score, the number of sleep strategies used increases by 0.10 (*p* ≤ 0.0001). This analysis had a *R* ^2^ value of 7.6%. The linear regression using both dichotomised and numerical BIS values is shown in Figure 1. When looking at the type of strategies used, we saw some differences in people with and without insomnia. Insomnia was associated with a higher prevalence of using sleep medications and smoking, and a lower prevalence of following a routine and having sex with a partner (Data S1, Table S3).

### 3.4 Music for sleep

**FIGURE 2:**
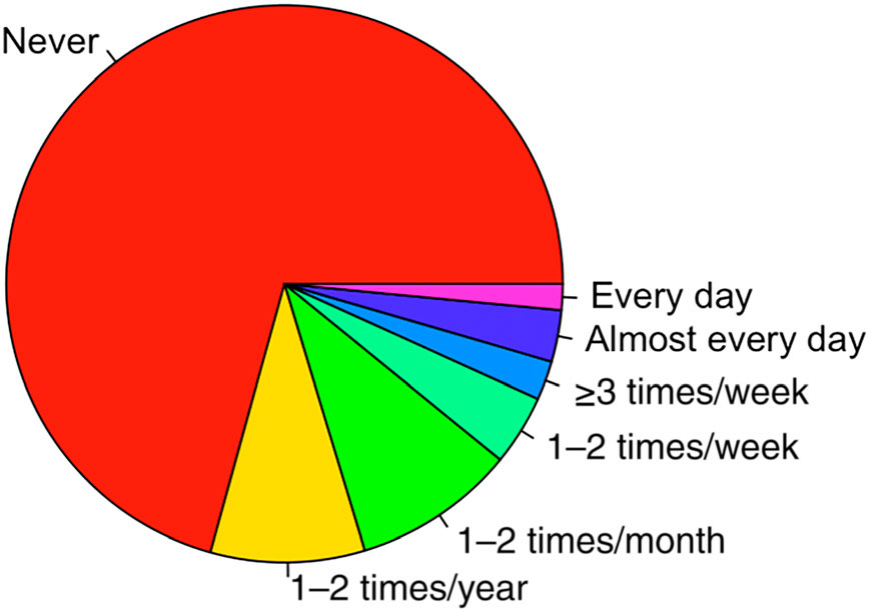
Prevalence of music use for sleep. Music was never used as a sleep aid by 70.7% of the participants. In all, 9% used music 1–2 times/year, whereas 9.5% used music 1–2 times/month. Some of the participants used music on a weekly basis with 4.1% using music 1–2 times/week, 2.3% using music ≥3 times/week, 3% using music almost every day, and 1.5% using music daily.

When evaluating the use of music as a strategy for improving sleep, we found that 20% of participants used music as a sleep aid at least 1–2 times/month, while 11% used music at least 1–2 times/week, and 4.5% used music for sleep either every day or almost every day. Figure 2 shows a pie chart of how the frequency of music use is distributed.

Using the chi‐square association test, we found that the use of music for sleep was significantly more common among younger people (*p* = 0.00025), people who had insomnia (*p* = 0.00021) and people who had sleep problems (*p* = 0.00013). The initial explorative chi‐square test also indicated associations between the use of music and both educational and income level (Table 3). Analysis of music engagement among participants using music for sleep, as measured by the Gold‐MSI active engagement subscale (Møller et al., 2024), revealed a mean (standard deviation [SD]) score of 3.54 (1.2), significantly lower than the normative data for age‐ and gender‐matched individuals in the Danish population (mean [SD] 4.39 [1.02]) (Møller et al., 2024) (*p* < 0.0000001). This suggests that individuals who use music for sleep engage with it less actively, potentially in a more passive or functional manner. As music engagement was only assessed among music users, we cannot compare it to engagement levels within the broader study sample.

**TABLE 3:** Chi‐square association test between the use of music for sleep and sociodemographic variables, insomnia, and sleep problems.

Variable	No, *n* (%)	Yes, *n* (%)	*χ* ^2^ (df)	*p* ^a^
Sex			2.0 (1)	0.16
Male	459 (78)	130 (22)		
Female	493 (81)	113 (19)		
Age (years)			23.7 (5)	0.00025
18–29	161 (69)	71 (31)		
30–39	146 (79)	39 (21)		
40–49	142 (78)	40 (22)		
50–59	171 (84)	32 (16)		
60–69	145 (85)	26 (15)		
≥70	187 (84)	35 (16)		
Education (Danish levels of education)			31.3 (5)	0.0000082
Primary school	22 (67)	11 (33)		
USE	81 (64)	46 (36)		
VE	149 (79)	40 (21)		
Bachelor	347 (81)	81 (19)		
Master	297 (83)	59 (17)		
PhD	56 (90)	6 (10)		
Income (DKK)^b^			39.7 (6)	0.00000053
<100,000	71 (66)	37 (34)		
100,001–250,000	194 (77)	59 (23)		
250,001–400,000	222 (80)	56 (20)		
400,001–600,000	231 (87)	34 (13)		
600,001–800,000	86 (86)	14 (14)		
800,001–1,000,000	35 (81)	8 (19)		
>1,000,000	20 (95)	1 (5)		
Do not know	14 (54)	12 (46)		
Do not wish to answer	79 (78)	22 (22)		
Insomnia^c^			13.8 (1)	0.00021
No	641 (83)	132 (17)		
Yes	311 (74)	111 (26)		
Sleep problems^d^			14.7 (1)	0.00013
No	458 (85)	83 (15)		
Yes	494 (76)	160 (24)		

Abbreviations: df, degrees of freedom; DKK, Danish Kroner; USE, Upper Secondary Education; VE, Vocational Education.

*Note*: answers to the question: ‘What do you do to improve sleep?’, ‘Listening to music’ response option (‘Never’ and ‘1–2 times/year’ = no; ‘1–2 times/month’ ‘1–2 times/week’, ‘≥3 times/week’, ‘Almost every day’ and ‘Every day’ = yes).

^a^ Pearsons chi‐square test.

^b^ Chi‐square analysis is without the last two categories.

^c^ Measured using Bergen Insomnia Scale.

^d^ Measured using the Pittsburgh Sleep Quality Index.

The logistic regression test shown in Table 4 confirmed the associations indicated by the Pearson's chi‐squared test. The crude logistic regression showed a statistically significant relationship between age and listening to music for sleep, with older individuals being less likely to use music for sleep.

**TABLE 4:** Logistic regression analysis with listening to music for sleep as the dependent variable.

	Crude OR (95% CI)	Age‐adjusted OR (95% CI)	Adjusted OR^a^ (95% CI)
Sex			
Male	1.0 (ref.)	1.0 (ref)	1.0 (ref)
Female	0.81 (0.61–1.07)	0.82 (0.62–1.09)	0.76 (0.56–1.02)
Age (years)			
18–29	1.0 (ref)	‐	1.0 (ref)
30–39	**0.61 (0.38–0.95)**	‐	0.74 (0.46–1.20)
40–49	0.64 (0.41–1.00)	‐	0.82 (0.50–1.33)
50–59	**0.42 (0.26–0.67)**	‐	**0.55 (0.33–0.90)**
60–69	**0.41 (0.24–0.66)**	‐	**0.50 (0.29–0.85)**
≥70	**0.42 (0.27–0.67)**	‐	**0.49 (0.30–0.79)**
Education (Danish levels of education)			
Primary school	1.0 (ref)	1.0 (ref)	1.0 (ref)
USE	1.14 (0.51–2.63)	0.92 (0.41–2.17)	0.93 (0.41–2.21)
VE	0.54 (0.24–1.23)	0.62 (0.28–1.45)	0.62 (0.28–1.46)
Bachelor	0.47 (0.22–1.04)	0.48 (0.22–1.07)	0.51 (0.24–1.15)
Master	**0.40 (0.19–0.89)**	**0.39 (0.18–0.88)**	**0.43 (0.20–0.98)**
PhD	**0.21 (0.07–0.63)**	**0.23 (0.07–0.68)**	**0.24 (0.07–0.72)**
Income (DKK)			
<100,000	1.0 (ref)	1.0 (ref)	‐
100,001–250,000	**0.58 (0.36–0.96)**	0.75 (0.44–1.29)	‐
250,001–400,000	**0.48 (0.30–0.80)**	0.63 (0.36–1.10)	‐
400,001–600,000	**0.28 (0.16–0.48)**	**0.34 (0.18–0.64)**	‐
600,001–800,000	**0.31 (0.15–0.61)**	**0.40 (0.18–0.84)**	‐
800,001–1,000,000	0.44 (0.17–1.00)	0.53 (0.20–1.31)	‐
>1,000,000	**0.10 (0.01–0.49)**	**0.11 (0.01–0.59)**	‐
Do not know	1.64 (0.68–3.93)	1.96 (0.79–4.82)	‐
Do not wish to answer	**0.53 (0.29–0.98)**	0.76 (0.38–1.52)	‐
Insomnia BIS			
No	1.0 (ref)	1.0 (ref)	1.0 (ref)
Yes	**1.73 (1.30–2.31)**	**1.75 (1.31–2.34)**	**1.68 (1.24–2.26)**
PSQI sleep problems			
No	1.0 (ref)	1.0 (ref)	‐
Yes	**1.79 (1.34–2.41)**	**1.84 (1.37–2.49)**	‐

Abbreviations: BIS, Bergen Insomnia Scale; CI, confidence interval; DKK, Danish Kroner; OR, odds ratio; PSQI, Pittsburgh Sleep Quality Index; USE, Upper Secondary Education; VE, Vocational Education.

*Note*: answers to the question: ‘What do you do to improve sleep?’, ‘Listening to music’ response option (‘Never’ and ‘1–2 times/year’ = 0; ‘1–2 times/month’ or more = 1). Statistically significant values are highlighted in bold.

^a^ The adjusted model includes sex, age, education and insomnia. Income is not included in the adjusted model as it is most likely highly confounded with age.

Furthermore, the logistic regression showed a significant positive association between using music for sleep and having insomnia, with an OR of 1.73 (95% confidence interval [CI] 1.30–2.31) in the crude model and 1.68 (95% CI 1.24–2.26) in the adjusted model. This means that participants with insomnia are 1.68 times more likely to use music for sleep than people without insomnia after adjusting for the potential influence of age, gender, and educational level. A statistically significant relationship between using less music and having a higher education level was persistent through different adjustment models. The crude logistic regression showed a statistically significant association between listening to music for sleep and income level, which became weaker when adjusting for more variables.

## 4 DISCUSSION

### 4.1 Summary of findings

This study aimed to map the strategies used for sleep in the general population and found that 99% of the participants used at least one strategy 1–2 times/month. The most common strategies included following a routine, reducing caffeine in the evening/afternoon, and lowering the temperature in the bedroom. In accordance with our hypothesis, we found that people with insomnia used significantly more strategies to improve their sleep than people without insomnia. Our results showed that 20% of participants used music to help them sleep at least 1–2 times/month, while only 11% of participants used music on a weekly basis. People using music for sleep tended to be younger and have insomnia. These results give a comprehensive view of the nature and frequency of sleep strategies used in the general population.

### 4.2 Sleep strategies

We found that 99% of the population used at least one strategy to improve their sleep at least 1–2 times/month. Thus, even though our results support the hypothesis that individuals with insomnia tend to employ more sleep strategies (Bjorvatn, Waage, & Saxvig, 2023; Morin et al., 2006), we also show that people without insomnia consider their behaviour in terms of how it may facilitate good sleep. On average, people without insomnia used 6.6 strategies at least 1–2 times/month, reflecting a broad spectrum of strategies used. A Norwegian study used a similar approach and found that only 34.3% of participants used a method or trick to fall sleep (Bjorvatn, Waage, & Saxvig, 2023). This difference is quite notable given that both studies focused on sleep strategies in a population sample of neighbouring countries with similar socioeconomic status. However, a number of differences in the approach may explain this gap. First, the study by Bjorvatn et al. (Bjorvatn, Waage, & Saxvig, 2023) focused on sleep initiation, whereas our study focused on sleep in general. Second, an important difference between the studies is the way the question was phrased. Bjorvatn et al. (Bjorvatn, Waage, & Saxvig, 2023) asked *if* people used a method or trick to sleep, and only when people responded ‘yes’, the strategies were presented with a binary ‘yes/no’ response option. In the present study, we included an open question (‘What do you do to improve your sleep?’) with response options presented in a 7‐point Likert scale ranging from ‘Never’ to ‘Every day’, which allowed for the assessment of the prevalence of each strategy, and made all participants consider their use of each strategy. Another important difference is the number of strategies included. In our study, we took a comprehensive approach addressing a broad range of strategies suggested in the literature and evaluated in a pilot experiment. In total, we included 24 strategies, ranging from general behaviour, such as following a routine, to specific behaviours, such as listening to music or doing breathing exercises. In contrast, the study by Bjorvatn et al. (Bjorvatn, Waage, & Saxvig, 2023) presented seven strategies and an ‘other’ option. None of our three most common strategies (following a routine, avoiding afternoon caffeine, and lowering bedroom temperature) were included as response options in the Bjorvatn et al. study (Bjorvatn, Waage, & Saxvig, 2023). Taken together, these variations may explain why our results show a high use of sleep strategies compared to the study by Bjorvatn et al. (Bjorvatn, Waage, & Saxvig, 2023).

The fact that we asked about frequency of use means that our results differentiate between strategies used on a monthly, weekly or daily basis. A study performed on Canadian university students used an similar approach and showed that in this young sample, some strategies tended to be used often (such as reading, adjusting the heat and watching television), whereas people who used strategies such as sleep medications, writing diary or sexual activity usually did so on a less regular basis (Brown et al., 2017). Similarly, our results showed that the popular strategies of following a regular routine, reducing caffeine and lowering the temperature were used by many on a daily basis, whereas other strategies that many people used on a monthly basis, such as exercise, sexual activity and breathing exercises, were rarely used daily. As such, the results of the present study provide more detail both on the number and type of strategies used, and on which types of strategies are used regularly or occasionally.

### 4.3 Sleep strategies and insomnia

Our results showed that people with insomnia tended to use more strategies than people without insomnia. However, the study is not designed to determine causality, and we need to be careful in the interpretation. It is not yet clear whether individuals with insomnia use more strategies because their sleep is worse, or if their sleep quality is worsened by the use of multiple strategies. Still, we know from previous research that some sleep strategies are beneficial for sleep (e.g., reducing blue light exposure) whereas other strategies can have a negative impact on sleep (e.g., using a phone with blue light) (Silvani et al., 2022). In the present study, we found that people with insomnia utilised both positive (e.g., CBT‐I and sleep hygiene elements) and potentially negative strategies more (Data S1, Table S3), suggesting a complex relationship where insomnia may drive the use of both positive and negative strategies. Interestingly, when adjusting for the overall higher use of strategies in people with insomnia, we saw that people with insomnia were less likely to follow a routine to improve sleep, but more likely to smoke cigarettes and use sleep medications (Data S1, Table S3). This shows that many people still turn toward pharmacological solutions for insomnia, despite CBT being recommended as first‐line treatment. Individuals with insomnia may lack awareness or guidance about which strategies are most effective. This highlights the need for targeted education and interventions to promote evidence‐based, beneficial sleep strategies while discouraging counterproductive ones. Clinicians and public health initiatives could play a key role in steering individuals toward healthier sleep behaviours.

### 4.4 Music users

One of the sleep strategies reported in several studies is listening to music. By zooming in on this strategy, we found support for the hypotheses that individuals who use music for sleep are younger and more likely to have insomnia (Aritake‐Okada et al., 2009; Bjorvatn, Waage, & Saxvig, 2023; Trahan et al., 2018). Our observed yearly prevalence of 29% of people using music to sleep is consistent with findings from a Canadian population study showing that 25.2% of the population had used music for sleep within the last year (Morin et al., 2006). Other previous studies have grouped listening to music with reading (Aritake‐Okada et al., 2009; Urponen et al., 1988), making the results harder to compare. A study from the UK found that 62% of respondents used music as sleep aid at least once (Trahan et al., 2018), thus finding a significantly higher prevalence of music use as a sleep aid than our study did. However, this study was not representative of the British population, as it was specifically addressing music and sleep, presumably resulting in a sampling bias, where people using music for sleep were more likely to participate.

Our study adds to the current knowledge by collecting data in a population sample, not only on whether people use music for sleep or not, but also on the frequency of use. One previous study in Canadian university students used a similar approach and found that even though music was used as a sleep strategy by 55% of the sample, only 17% used music as a sleep aid weekly (Brown et al., 2017). As such, this study showed that 31% of those who used music as a sleep strategy used music on a weekly basis. In our results, 37% of music users, used music on a weekly basis. As such, our results suggest that even though a substantial amount of the population listen to music for sleep sometimes, it is much less common to use music as sleep aid on a weekly or even daily basis. As mentioned above, the most popular strategies seemed to be used more frequently. For example, the strategy ‘following a routine’ was used by 884 of the participants and 833 of these reported using this strategy on a weekly basis (94%). Other strategies like ‘listening to podcast’ are more similar to music and show similar prevalence and frequency. Even though podcasts are also an auditory stimulus they are different in terms of structure and acoustic features (Dittrich, 2023), and there seem to be no studies yet on the effect of listening to podcast on sleep. Related research has compared the effects of music and audiobooks on sleep and found that music was more efficient for improving sleep (Harmat et al., 2008; Jespersen et al., 2019).

In the study among the Canadian university students 55% reported using music for sleep (Brown et al., 2017). This is in line with our findings showing a higher prevalence of using music as a sleep aid among younger participants. Still, it is not clear from our study if the higher prevalence of using music for sleep in younger participants is a true age effect or if it is rather a generation effect. Music has become increasingly accessible in recent years through online streaming services providing 24‐h access to music (IFPI, 2019). The younger generations are therefore growing up with a larger access to music and may hence use it more for specific behavioural purposes such as sleep. Whether this is a result of age or rather a cultural habit that the younger generation will maintain throughout the lifespan remains to be seen.

### 4.5 Strengths and limitations

This study had the strength of being representative regarding age groups and sex, and of having a large sample size of 1195 participants, allowing for more statistical power. Nonetheless, the study had limitations that should be considered in the interpretation of the results. One limitation is the potential for sampling bias. The use of Meta advertisements to distribute the survey, allowed for a wider reach of the survey. However, the focus on sleep strategies in the advertisement may have attracted participants with sleep difficulties. Comparing the sleep and insomnia scores suggests that this may be the case. The mean (SD) PSQI score of 6.56 (3.35) is vaguely higher than in other non‐clinical populations (Mollayeva et al., 2016). The BIS scores indicated that 35% of our subsampled population had insomnia. This result is higher than the recent findings of 24.9% among the general Norwegian population using the same scale (Bjorvatn, Waage, & Saxvig, 2023). It is also important to keep in mind that even though the BIS is well‐validated (Pallesen et al., 2008), there is a risk that self‐report questionnaires overestimate insomnia disorder compared to clinical assessment. In survey studies, people may base their report on recent transient symptoms, or the insomnia symptoms may be better explained by other sleep disorders not recognised by the respondent (Bjorvatn et al., 2021). Another limitation is the potential variation in how people respond to the question on sleep strategies. Some participants may report their deliberate sleep strategies, while others report more incidental behaviours like smartphone use. Additionally, certain activities like exercise and reading a book may serve multiple purposes, with sleep improvement being a more or less subconscious factor. Still, the pilot testing of the phrasing of the survey indicated that people of various ages understood the question well.

## 5 CONCLUSION

By evaluating a broad range of sleep strategies, this study shows that people do several things to promote sleep in their daily lives. We found that insomnia is related to using more sleep strategies, but even people without insomnia consider specific behaviours to facilitate sleep. Music is commonly used as a sleep aid by 11% of the Danish population and the use of music as a sleep strategy was associated with insomnia and associated with a younger age. By mapping the sleep strategies in the general population, this research enhances our understanding of various sleep behaviours and contributes to the evidence‐base regarding sleep strategies. This knowledge provides an important foundation for future public health interventions aiming to improve sleep in the general population by minimising sleep disruptive behaviours and enhancing positive sleep strategies instead.

## AUTHOR CONTRIBUTIONS

**Rasmus Møller Buus:** Investigation; formal analysis; visualization; writing – original draft. **Silvia Genovese:** Investigation; visualization; writing – review and editing. **Kira Vibe Jespersen:** Conceptualization; funding acquisition; methodology; project administration; supervision; writing – review and editing.

## FUNDING INFORMATION

The project was implemented with financial support from Trygfonden (identification number: 178123). The salary of Silvia Genovese is covered by funding from European Union Marie Curie Doctoral Network ‘Lullabyte’.

## CONFLICT OF INTEREST STATEMENT

The authors declare no conflicts of interest.

## Supporting information


**Data S1**Supporting Information.

## Data Availability

The data that support the findings of this study are available on request from the corresponding author. The data are not publicly available due to privacy or ethical restrictions.
