# Supplementary material for: The art of sleep: examining sleep strategies in the general population with a focus on the use of music for sleep
Source: J Sleep Res. 2025 Feb 19;34(5):e70006. doi: 10.1111/jsr.70006 (PMC12426710; doi:10.1111/jsr.70006)
Supplement: Supplementary file 1 — Data S1Supporting Information. [file JSR-34-e70006-s001.pdf]

Supplementary material

The Art of Sleep: Examining sleep strategies in the general population with a focus on the use of music for sleep

Rasmus Møller Buus[1], Silvia Genovese[2], Kira Vibe Jespersen[2*]

Content:

Supplementary Table S1 Contents of the survey

Supplementary Table S2 Sleep strategies prevalence

Supplementary Table S3 The type of sleep strategies used by individuals with insomnia compared to those without

Supplementary Figure S1 Illustration of subsampling

Supplementary Figure S2 Geographical distribution of respondents

Supplementary Table S1(a)
Contents of the Survey - demographic variables and sleep scales.

Demographic variables		
**Variable**	**N. of questions**	**Sub-categories**
Age	1	
Sex	1	
Municipality of residence	1	
Socio-economic factors	2	Education
		Income
Nationality	1	
**Sleep scales**		
**Variable**	**N. of questions**	**Scale**
Sleep Quality	20	Pittsburgh Sleep Quality Index (PSQI)[1]
Insomnia	6	Bergen Insomnia Scale (BIS)[2]

Supplementary Table S1(b)
Contents of the survey – sleep strategies

Sleep strategies[3, 4]	
**Suggested sleep strategies**	**Type of question**
Reading a book	7-point Likert scale from “Never” to “Everyday”
Listening to music	
Listening to podcast	
Lowering temperature in the bedroom	
Watching TV/streaming	
Taking a hot shower	
Doing meditation/mindfulness	
Doing body scan	
Doing breathing exercises	
Listening to white noise	
Using non-prescription sleep medication	
Using prescription sleep medication	
Having sex with a partner	
Having sex alone/masturbation	
Writing in a diary	
Drinking alcohol	
Smoking cigarettes	
Using a sleep mask	
Doing exercise	
Using a phone (scrolling, reading, gaming)	
Following a routine	
Reducing caffeine in the afternoon/evening	
Reducing blue light exposure	
Praying	
Other, please specify	

Supplementary Table S1(c)
Contents of the Survey – music

Music preferences and motivations		
**Variable**	**N. of questions**	**Sub-categories**
Music Preference	3	Sleep music preferences[4]
		General music preferences
		Timing of listening to music for sleep
Music Motivations	2	"How" music aids sleep
		"Why" they choose to listen to music for sleep
**Music scales**		
**Variable**	**N. of questions**	**Scale**
Music Engagement	9	Goldsmith Musical Sophistication Index (Gold-MSI) – Active Engagement subscale[5]
Musical Training	1	Ollen Musical Sophistication Index [6]

Table S1(c) is only presented to those, who respond that they use music for sleep (at any frequency).

Supplementary Table S2

Sleep strategy prevalence: yearly, monthly, weekly, daily

Sleep strategy	Used at all (yearly)[a]	Used at least 1-2 times per month	Used at least 1-2 times per week	Used almost every day/every day
Following a routine	884 (74%)	868 (73%)	833 (70%)	644 (54%)
Reducing caffeine in the afternoon/evening	791 (66%)	780 (65%)	757 (63%)	676 (57%)
Lowering temperature in the bedroom	775 (65%)	739 (62%)	665 (56%)	560 (47%)
Using a phone (scrolling, reading, gaming)	662 (55%)	636 (53%)	544 (46%)	302 (25%)
Reading a book	775 (65%)	627 (52%)	445 (37%)	257 (22%)
Reducing blue light exposure	550 (46%)	521 (44%)	481 (40%)	384 (32%)
Having sex alone / masturbation	618 (52%)	514 (43%)	242 (20%)	49 (4%)
Doing exercise	518 (43%)	484 (41%)	395 (33%)	77 (6%)
Watching TV or streaming	447 (37%)	409 (34%)	329 (28%)	176 (15%)
Doing breathing exercises	504 (42%)	377 (32%)	206 (17%)	71 (6%)
Having sex with a partner	467 (39%)	367 (31%)	152 (13%)	6 (1%)
Doing meditation or mindfulness	349 (29%)	260 (22%)	134 (11%)	53 (4%)
Listening to podcast	286 (24%)	247 (21%)	176 (15%)	110 (9%)
Listening to music	350 (29%)	243 (20%)	130 (11%)	54 (5%)
Drinking alcohol	321 (27%)	235 (20%)	133 (11%)	31 (3%)
Other, please specify	238 (20%)	231 (19%)	210 (18%)	163 (14%)
Taking a hot shower	317 (27%)	224 (19%)	115 (10%)	32 (3%)
Praying	208 (17%)	170 (14%)	119 (10%)	86 (7%)
Doing body scan	263 (22%)	162 (14%)	74 (6%)	21 (2%)
Using non-prescription sleep medication	158 (13%)	112 (9%)	73 (6%)	33 (3%)
Using prescription sleep medication	137 (11%)	106 (9%)	79 (7%)	49 (4%)
Using a sleep mask	137 (11%)	100 (8%)	70 (6%)	41 (3%)
Smoking cigarettes	91 (8%)	83 (7%)	73 (6%)	55 (5%)
Listening to white noise	112 (9%)	77 (6%)	39 (3%)	20 (2%)
Writing in a diary	116 (10%)	72 (6%)	40 (3%)	23 (2%)

Note. Participants were allowed to select more than one option.

[a] was used to sort responses from most to least popular.

Respondents were presented with the response options “Never”, “1 – 2 times a year”, “1 – 2 times a month”, “1 – 2 times a week”, “3 times a week”, “Almost every day” and “Every day”.

Supplementary Table S3

The type of sleep strategies used by individuals with insomnia compared to those without

Sleep strategies	OR (95% CI)	Adjusted for general number of strategies used
Following a routine	0.97 (0.92-1.02)	**0.91 (0.86-0.96)**
Reducing caffeine in the afternoon/evening	**1.11 (1.06-1.16)**	NS
Lowering temperature in the bedroom	**1.07 (1.02-1.12)**	NS
Using a phone (scrolling, reading, gaming)	**1.12 (1.07-1.18)**	NS
Reading a book	1.01 (0.95-1.06)	NS
Reducing blue light exposure	**1.13 (1.08-1.18)**	NS
Having sex alone / masturbation	**1.16 (1.07-1.26)**	NS
Doing exercise	**1.14 (1.06-1.21)**	NS
Watching TV or streaming	**1.08 (1.02-1.14)**	NS
Doing breathing exercises	**1.22 (1.13-1.31)**	NS
Having sex with a partner	0.99 (0.90-1.10)	**0.85 (0.76-0.94)**
Doing meditation or mindfulness	**1.10 (1.01-1.19)**	NS
Listening to podcast	**1.12 (1.05-1.20)**	NS
Listening to music	**1.16 (1.07-1.26)**	NS
Drinking alcohol	**1.13 (1.04-1.24)**	NS
Other, please specify	1.04 (0.98-1.10)	NS
Taking a hot shower	**1.11 (1.01-1.21)**	NS
Praying	1.03 (0.95-1.11)	NS
Doing body scan	**1.22 (1.09-1.36)**	NS
Using non-prescription sleep medication	**1.40 (1.25-1.57)**	**1.28 (1.15-1.44)**
Using prescription sleep medication	**1.40 (1.27-1.56)**	**1.32 (1.20-1.47)**
Using a sleep mask	1.02 (0.92-1.12)	NS
Smoking cigarettes	**1.18 (1.08-1.29)**	**1.14 (1.04-1.25)**
Listening to white noise	**1.18 (1.04-1.35)**	NS
Writing in a diary	**1.14 (1.01-1.30)**	NS

Note. Logistic regression test with insomnia as the dependent variable. OR are the odds of having insomnia associated with a one-unit increase of frequency of utilizing the sleep strategy.

NS = non-significant

Supplementary Figure S1: Illustration of subsampling

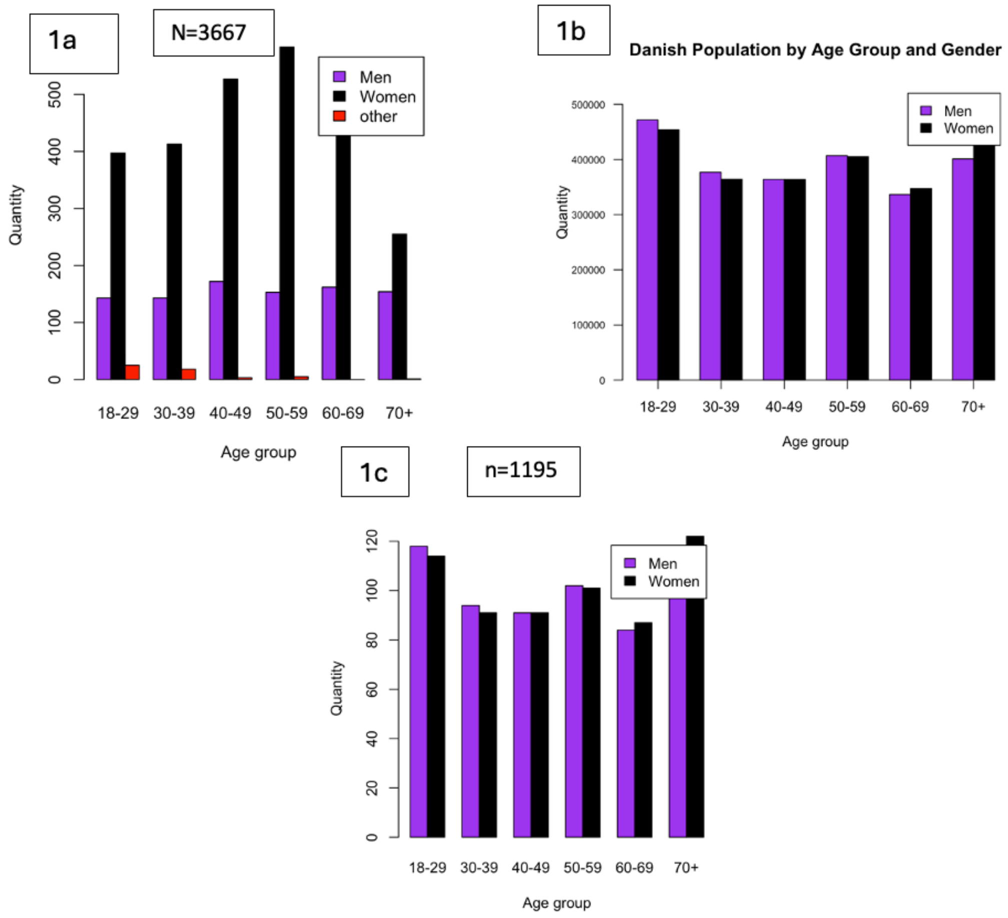

Supplementary Figure S1 Illustration of subsampling. Age and sex distribution of a) the original dataset, b) the Danish population and c) the subsample of the dataset.

Supplementary Figure S2: Geographical distribution of respondents

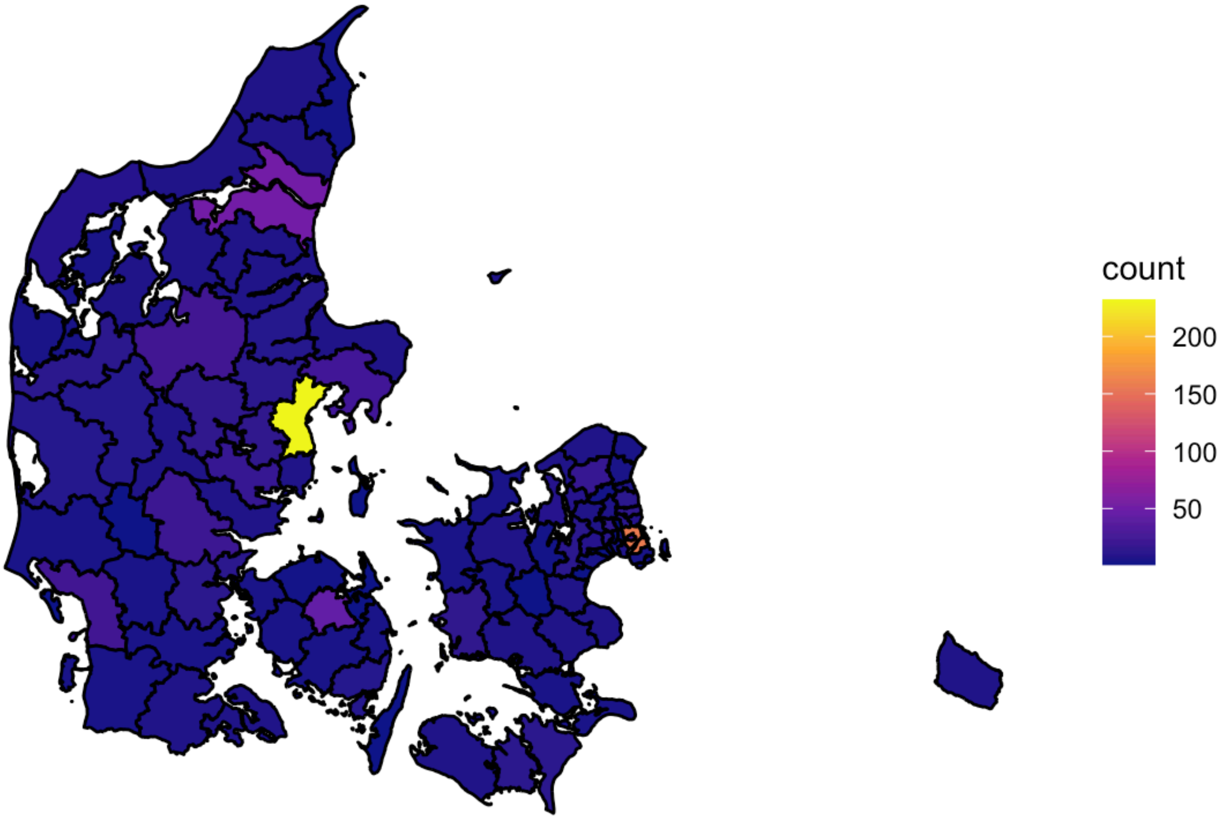

Supplementary Figure S2 Geographical distribution of respondents across the 98 municipalities. The study included respondents from all 98 municipalities with the most respondents from Aarhus (232), Copenhagen (157), Aalborg (52) and Odense (44), corresponding to the 4 largest cities of Denmark.

References

1. Buysse, D.J., et al., *The Pittsburgh Sleep Quality Index: a new instrument for psychiatric practice and research.* Psychiatry research, 1989. **28**(2): p. 193-213.
2. Pallesen, S., et al., *A New Scale for Measuring Insomnia: The Bergen Insomnia Scale.* Perceptual and Motor Skills, 2008. **107**(3): p. 691-706.
3. Brown, C.A., P. Qin, and S. Esmail, *“Sleep? Maybe later…” A cross-campus survey of university students and sleep practices.* Education Sciences, 2017. **7**(3): p. 66.
4. Trahan, T., et al., *The music that helps people sleep and the reasons they believe it works: A mixed methods analysis of online survey reports.* PloS one, 2018. **13**(11): p. e0206531.
5. Müllensiefen, D., et al., *The musicality of non-musicians: An index for assessing musical sophistication in the general population.* PloS one, 2014. **9**(2): p. e89642.
6. Ollen, J.E., *A criterion-related validity test of selected indicators of musical sophistication using expert ratings*. 2006, The Ohio State University.
